# Heart failure prognostic model

**Published:** 2011-05-25

**Authors:** L Axente, C Sinescu, G Bazacliu

**Affiliations:** * Department of Cardiology, ‘Bagdasar–Arseni’ Emergency Hospital, BucharestRomania; ** Faculty of Power Engineering, Polytechnic University of BucharestRomania

**Keywords:** heart failure, Cox multiple regression model, survival function, prognosis

## Abstract

Heart failure (HF) is a common, costly, disabling and deadly syndrome. Heart failure is a progressive disease characterized by high prevalence in society, significantly reducing physical and mental health, frequent hospitalization and high mortality (50% of the patients survive up to 4 years after the diagnosis, the annual mortality varying from 5% to 75%).

The **purpose** of this study is to develop a prognostic model with easily obtainable variables for patients with heart failure.

**Methods and Results**. Our lot included 101 non–consecutive hospitalized patients with heart failure diagnosis. It included 49,5% women having the average age of 71.23 years (starting from 40 up to 91 years old) and the roughly estimated period for monitoring was 35.1 months (5–65 months).

Survival data were available for all patients and the median survival duration was of 44.0 months. A large number of variables (demographic, etiologic, co morbidity, clinical, echocardiograph, ECG, laboratory and medication) were evaluated. We performed a complex statistical analysis, studying: survival curve, cumulative hazard, hazard function, lifetime distribution and density function, meaning residual life time, Ln S (t) vs. t and Ln(H) t vs. Ln (t). The Cox multiple regression model was used in order to determine the major factors that allow the forecasting survival and their regression coefficients: age (0.0369), systolic blood pressure (–0.0219), potassium (0.0570), sex (–0.3124) and the acute myocardial infarction (0.2662).

**Discussion**. Our model easily incorporates obtainable variables that may be available in any hospital, accurately predicting survival of the heart failure patients and enables risk stratification in a few hours after the patients' presentation. Our model is derived from a sample of patients hospitalized in an emergency department of cardiology, some with major life–altering co morbidities. The benefit of being aware of the prognosis of these patients with high risk is extremely beneficial. The use of this model may ease the estimation of the vital prognosis, to improve the compliance and increase in the use of life–saving medical or surgical therapy (pacemakers, implantable defibrillators or transplantation).

## Introduction

Heart failure (HF) is a common, costly, disabling, and deadly [1] syndrome. HF is a syndrome in which a structural or a functional cardiac condition impairs the heart's ability to provide sufficient blood flow in order to meet the body's needs, or to do that at an elevated diastolic pressure [2]. Heart failure is a progressive disorder characterized by high prevalence in society, significantly reduced physical and mental health [3,4] –reduced quality of life, frequent hospitalization and high mortality (50% of the patients survive for 4 years after diagnosis [5], the annual mortality varying from 5% to 75%).

Until now, the focus was to define the heart failure, the descriptive terms and its treatment. No system of diagnostic criteria has been agreed as the gold standard for the heart failure. There were many definitions of this complex syndrome, but none was satisfactory, due to a lack of a universally agreed definition and to the challenges in the definitive/peremptory diagnosis. The commonly used systems were the ‘Framingham criteria’ [6] (derived from the Framingham Heart Study), the ‘Boston criteria’ [7], the ‘Duke criteria’ [8], and (in the setting of acute myocardial infarction) the ‘Killip class’ [9]. 

The new American and European guidelines and recommendations include new information and have the declared intention to simplify and clarify the previous recommendations [5]. Heart failure is a clinical syndrome in which patients have features, symptoms typical to heart failure and signs typical to heart failure and objective evidence of a structural or functional abnormality of the heart at rest [5]. 

Classification of heart failure [5]:

- New onset: First presentation, Acute or slow onset
- Transient: Recurrent or episodic
- Chronic: Persistent, Stable, worsening, or decompensated

In recent years, the emphasis has been on morbidity, quality of life, costs, prognosis and mortality. In terms of incidence, prevalence, morbidity and mortality, the epidemiologic magnitude of heart failure (HF) is staggering. 

Heart failure is a major public healthcare problem reducing the quality of life, and, has a considerable economic cost to the individual and the society in general. This ‘cardiovascular epidemic’ affects over 5 million people in the U.S., approximately 2% of adults and 10% of the elder people [10]. With an incidence of over 400,000 new cases diagnosed each year [11], and approximately 1 million hospital admissions annually, out of which over 80%  patients are aged over 65 years [11, 12], the heart failure is the only major cardiovascular disease that increases in the United States. Today, it is estimated that the prevalence in the European countries ranges between 0.4 and 2% [13], the studies showing that about 14 million of the approximately 900 million inhabitants of the 51 European countries, suffer from heart failure. Recent data from Statistics by Country for congestive Heart Failure [14] estimate an incidence of 0.146% (32 875) cases of heart failure and a prevalence of 1.76% (394 509) cases of heart failure for Romania. The President of the Romanian Heart Failure Working Group estimates the number of heart failure patients at approximately 800–900 000, a number permanently increasing [15].

HF morbidity is particularly important, because the patients with heart failure require frequent medical visits at home or re–hospitalization, representing a significant expenditure of health resources. In the first year after hospital discharge, approximately 50% of the patients with heart failure require re–hospitalization and the data are similar in those with systolic dysfunction or diastolic dysfunction.

Some European studies show that about 1% of the national health budget is allocated to the therapy of heart failure, while in the U.S., about 2% of the national health budget is allocated to this problem. The United States spends about dollar 20 billion annually, 10% of healthcare budget allocated for the management of cardiovascular disease with heart failure, 75% of the amount being allocated to hospital care. Heart failure is the most expensive cardiological syndrome [16, 17].

The prognosis of individual patients differs considerably, outcomes in highly variable trial data and it often does not give an adequate direction. Taking into consideration the magnitude of this syndrome in the society and its complexity, we need a model to predict the risk of death, to estimate the survival of heart failure patients. 

Prognosis is heterogeneous and depends on a large number of predictors. Multiple studies and meta–analysis highlighted various predictive criteria, but the latest ESC guidelines summarize the knowledge about the conditions associated with a poor prognosis in heart failure:

**Table 1:** Conditions associated with a poor prognosis in heart failure–according to ESC guidelines 2008 [5]*Powerful predictors.

Demographics	Clinical	Electrophysiological	Functional/exertional	Laboratory	Imaging
Advanced age*	Hypotension*	Tachycardia Q waves	Reduced work, low peak VO2*	Marked elevation of BNP/NT pro–BNP*	Low LVEF*
Ischemic etiology	NYHA functional class III–IV*	Wide QRS*	Poor 6–minute walk distance	Hyponatraemia *	Increased LV volumes
Resuscitated sudden death*	Prior HF hospitalization	LV hypertrophy	High VE/VCO2 slope	Elevated troponin*	Low cardiac index
Poor compliance	Tachycardia	Complex ventricular arrhythmias*	Periodic breathing	Elevated biomarkers, neurohumoral activation	High LV filling pressure
Renal dysfunction	Pulmonary rales	Low heart rate variability		Elevated creatinine/BUN	Restrictive mitral filling pattern, pulmonary hypertension
Diabetes	Aortic stenosis	Atrial fibrillation		Elevated bilirubin	Impaired right ventricular function
COPD	Sleep–related breathing disorders			Elevated uric acid	
Depression					

Generally, the inpatient mortality rate for patients with heart failure is of 5–20%, while the outpatient mortality rate remains of 20% at the end of the first year post diagnosis and up to 50% at 5 years post diagnosis, despite marked improvement in medical and device therapy. Each re–hospitalization increases the mortality rate to 20–30%. The patients with acute heart failure have a very severe prognosis, large randomized trials with hospitalized patients for decompensated heart failure have shown a 9.6% mortality rate at 60 days and a 35.2% combined mortality rate and re–hospitalization at 60 days [18, 19]. Mortality is particularly high in patients with acute myocardial infarction associated with severe HF, with mortality close to 30% at 1 year [19, 20].

Existing models that predict the risk of death in heart failure patients have features that limit their medical practice applicability. These models relied on complex or invasive measures of cardiac performance [21]. 

The identification on the persons with the high risk of death might bring the increase of the use with respect to the appropriate means available, medical and surgical therapy or palliative care or psychological, psychosocial and spiritual needs of satisfaction to the healthcare providers.

The **purpose** of this study is to develop a prognostic model easy to use for patients with heart failure. This model incorporates multiple variables (easily obtainable) clinical, laboratory and medication; the variables used, some of them already proved, others not yet, concerning the association with the increased risk of death.

The incurred model aims to consider the simple variables that may be available in any hospital in order to risk stratify patients in a few hours after their presentation

Benefits expected- Using this model either by healthcare providers or patients can:

- facilitate the estimation of vital prognosis
- improve compliance
- monitor the effectiveness of drug therapies
- identify the medication that improves survival in order to increase its use

Ability to accurately assess the prognosis would allow clinicians:

- To advise patients about prognosis
- To sort patients
- To make decisions about medical or surgical therapy (pacemakers, implantable defibrillators or transplantation)

## Methods

### Patient Population

101 non–consecutive patients admitted in the Department of Cardiology, ‘Bagdasar–Arseni’ Emergency Hospital with diagnosis of heart failure, were included from January 2005 to April 2008. We screened all the patients admitted with I50.0 and I50.1 codes (ICD–10–CM codes – International Classification of Disease, 10th Edition, Clinical Modification) for primary or secondary diagnosis of heart failure. We included only patients who accepted the long term follow up by the first author of the article all of them trusty from the point of view of compliance to treatment and availability to come to the medical review. We excluded 17 patients with incomplete baseline data. The criteria for the case selection were ESC guidelines (2005). There was only one exclusion criteria to be included in the study – association with aortic stenosis and patients with any other heart failure etiology, age, EF, or co morbidities could be enrolled. 

### Data Collection

All the demographic and in–hospital outcome data were collected prospectively into a database. The author had full access to the data. Subsequent outcome and mortality data were obtained from long term follow up, including reviews of hospital records, outside records, telephone surveys. For this analysis, the primary outcome–major event was death, rather than device implantation or cardiac transplantation.

### Statistical Analysis

The database in place was de–identified. A series of clinical variables previously reported to be associated with mortality were evaluated. Variables examined were demographic data (age, sex), etiological (ischemic or not), co morbidity (diabetes, stroke, chronic obstructive pulmonary, cirrhosis, cancer, dementia), clinical (systolic blood pressure, heart rate, NYHA class at presentation, systemic or pulmonary congestion, atrial fibrillation, BMI), echocardiographic (ejection fraction, left ventricular end diastolic diameter), the ECG (QRS> 120ms), laboratory (hemoglobin, WBC, percentage lymphocytes, uric acid, lipid profile, serum ionograma , creatinine, urea, glucose) and medication (ACE inhibitors, beta blockers, statins, allopurinol, diuretics, antiplatelet, anticoagulant, nitrate, calcium blockers, digitalis, angiotensin–receptor blocker). The types of variables are integer, real or boolean. 

Univariate analysis was performed initially for the selected data. For each variable the following elements were studied: Kaplan Meier survival curve,  Kaplan Meier curves of the Nelson–Aalen estimator for H (t) – cumulative hazard and h (t) hazard function, the intensity of risk, lifetime distribution function (life extension) F(t) =Pr(T<t)	=1–S(t), and density function f(t) of lifetime distribution function, Mean Residual Life Time (mrl),  average remaining survival time given the population has survived beyond t0, plot of Ln S (t) vs. t and plot of Ln(H)  the natural logarithm of cumulative hazard vs. Ln (t).

Initially, the data were analyzed as a single group, than variables were analyzed based on the average, median, specially chosen values and in several subgroups.

The key element of interest of this study is the survival function S defined as: S(t)=Pr(T>t) in which t represents one moment in time; T is a random variable designating the time (or age) of death; Pr( ) notes the probability of occurrence of an event. Survival analysis is a branch of statistics dealing with the life extension of biological organisms and involves the estimation of the survival until a certain event.

This study survival model uses hazard function logarithm. The parametric model of the hazard function logarithm, based on a multiple linear distribution, is: log h_i_(t)=alpha+a_1_x_i1_+a_2_x_i2_+...+a_n_x_in_ or h_i_(t)=exp(alpha+a_1_x_i1_+a_2_x_i2_+...+a_n_x_in_)= e^a_1_x_i1_+a_2_x_i2_+...+a_n_x_in_^

In which the index n is the number of independent variables noted xi1, xi2, …, xin index i represents the number of observation, the a1, a2,…, an, are the coefficients of the model, and alpha is the a baseline function. 

In order to establish the relationship between various variables, dependent or independent and survival, a multiple regression analysis was performed [22]. Cox multiple regression model [22], proportional hazards model was used for this analysis. The model h_i_(t)=h_0_e^a_1_x_i1_+a_2_x_i2_+...+a_n_x_in_^where  h_0_(t) is called baseline hazard function, it implies the survival function s(t)=exp(–H_0_(t)e^a^T^x^)=e^–H_0_(t)e^a^T^x^^in which  H_0_(t) is a cumulative hazard function, a is a n–dimensional column's vector of model parameters, with components a1, a2,…, an, x is a n–dimensional column's vector of independent variables with components x1  x2, …, xn , and T designates the sign of transposition of a vector. For baseline hazard function h_0_(t) respectively for H_0_(t) we used exponential known statistical models. [23].

## Results

One hundred one patients >18 years with diagnosis of heart failure were included. There were 50  (49,5%) women and 51 men included, with an average age of 71.23 years (40–91 years) followed up for an average of 35.1 months (5–65 months). Survival data were available for all patients and the median survival duration was of 44.0 months. During the follow–up 28, 54.9% of the men and 27, 54.4% of the women died. 

### The survival Analysis. Derivation of the Model

Initially, the data were analyzed as a single group then each variable was analyzed based on the average, median and in several subgroups with special chosen values.

**Figure 1:**
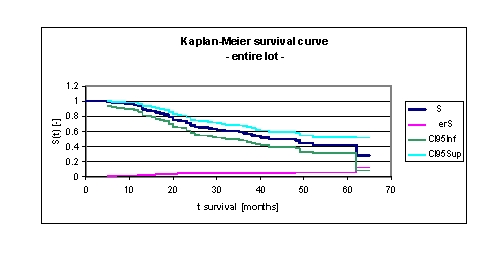
Kaplan–Meier survival curve for the entire lot

It was noted that for a random individual with heart failure, the probability of survival over t = 5 years is of 0,4231. Another interpretation of the values obtained is as it follows: 93,13% of the patients survive for one year, 70,8% survive for more than two years, 58,01% survive for more than three years, 47,95% survive for more than four years and only 42,31% of the population will survive for more than five years. It is noted that 49,8% of the patients survive for more than 44 months. 

**Figure 2:**
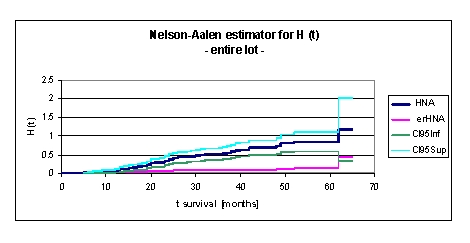
Nelson–Aalen estimator for H (t), cumulative hazard, for the entire lot

The cumulative hazard function is the integral of the hazard function. It can be considered as the probability of failure at time x given survival until time x. H(x)=∫ from –∞ to x h(μ)dμ

This might alternatively be expressed as H(x)=–ln(1–F(x). The cumulative hazard is the cumulative sum of the hazard. That means that the cumulative hazard at the failure time with rank K is the sum of the hazards of all failure times with ranks less than or equal to K.

We can see from the data obtained that H (t), cumulative hazard, for the entire lot increases from 0,0943 (at 1 year), 0,34 (2 years), 0,5373 (3 years), 0,6874 (4 years) to 0,8454 (5 years). It is noted that at 32 months H (t), cumulative hazard, is 0,5021.

**Figure 3:**
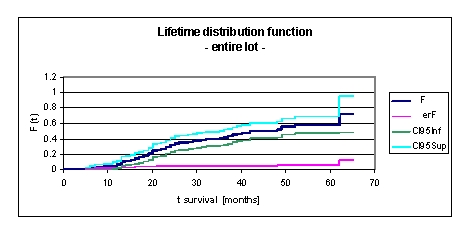
Lifetime distribution function F (t) –life extension, for the entire lot

Lifetime distribution function F (t) is defined by the relation F(t) =Pr(T<t)=1–S(t), where Pr (T < t) means the probability that death time T occurs before or at a specified time t, the latest.

In our lot, F (t) is monotonically increasing in time, this means that the likelihood of death increases as subject age increased. F (t) increasing in time from 0,0687 at 1 year, 0,292 at 2 years, 0,4092 at 3 years, 0,502 at 4 years to 0,5769 at 5 years. We can see from the data obtained, that in our lot, from 42 to 48 months, F (t) is constant 0,502, the likelihood of death being constant.

**Figure 4:**
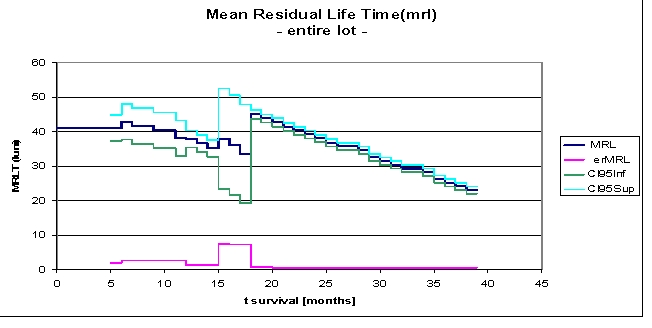
Mean Residual Life Time (mrl), for the entire lot

Mean Residual Life Time mrl (t0) is the average remaining survival time, given the population has survived beyond t0.

In our lot, mean residual lifetime is of 41.0802 months, given the patients have survived beyond 5 months, 38.0453 months at 12 months, 39.2295 months at 2 years and 26.2623 months, if the subjects in the study survived beyond 36 months. We note an increase of mean residual lifetime from 33.5869 to 44.9834 months at 18 months. After 39 months the data are not available, they are right censored.

**Figure 5:**
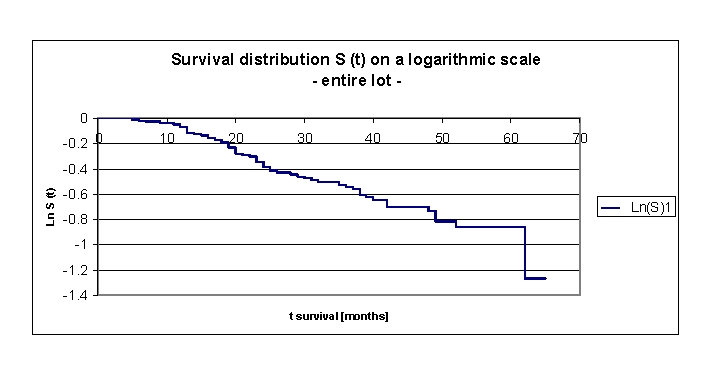
Survival distribution S (t) on a logarithmic scale, for the entire lot

It is useful to plot the survival distribution on a log scale. By doing so, we can identify the hazard rate as minus of the derivative of this function. A (approximate) straight line indicates that the exponential distribution may be a reasonable choice for the data. A non–straight line indicates that the exponential distributional assumption is not appropriate.

In our lot, survival distribution S (t) on a logarithmic scale is a straight line for the entire lot, these indicating that the choice of exponential distribution of other common parametric models is correct.

**Figure 6:**
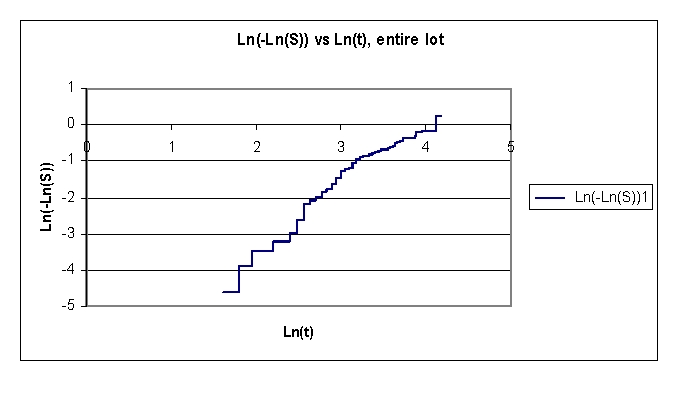
Plot of Ln(H)  the natural logarithm of cumulative hazard vs. Ln (t), for the entire lot

In our data set, the plot of log(–logS(t)) vs. logt is used to check if the Weibull model is a reasonable choice for the survival time given. The Weibull model is not a reasonable choice for the data. 

**Figure 7:**
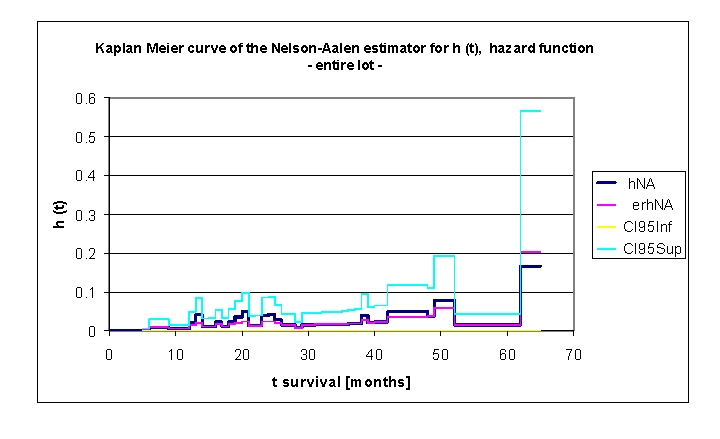
Kaplan Meier curve of the Nelson–Aalen estimator for h (t) hazard function, for the entire lot

An extremely important concept is the hazard function, conventionally denoted by h (t). Hazard function is the mortality rate at time t conditioned by survival until time t or later (T>t ). h (t) can be defined in mathematical terms as: h(t)=lim Δt–⟩ P(t≤T<t+ΔtΙT≥)/Δt=–S'(t)/S(t) in which S'(t)  represents the time derivative of survival function.

In our data set, we noted that the hazard function is not negative, this meaning that h(t)>0. The analysis of the data and the graph above denoted that the hazard function increasing and decreasing over time is non–monotonic in time. The appearance of ‘bathtub curve hazard function’ was noted; there were three such particular forms of the hazard function in the plot. The first curve can be described between 13 months (h (t)= 0.0206 and 0.0417) and 20 months (h (t)= 0.0482), the second between 24 months (h (t)= 0.0405) and 39 months (h (t)= 0.0396) and, the last one, between 49 months (h (t)= 0.0784) and 62 months (h (t)= 0.1667); finally, the rate of ‘wear out’ failures as the subjects exceed their lifetime design. The name is derived from the cross–sectional shape of a bathtub; the hazard function is large for small values of t, decreasing to some minimum and thereafter increasing again. This type of curve was initially described for technical systems, subsequently, the applicability being observed in survival analysis–a branch of statistics dealing with the life extent of biological organisms.

Other synonyms for hazard function are the force of mortality (particularly used in demography and actuarial science) or the hazard rate. The hazard function represents the ratio of the probability density function to the survival function, S(x).

Looking carefully in the de–identified and processed database, after ordering the patients, depending on the length of the life span, we were able to provide an explanation of the peaks of mortality recorded:

- at 13 months there were 4 deaths of patients > 74 years (74–79 years), three men and one woman, two with a Killip III acute myocardial infarction, a disabling myocardial infarction with low EF,
- at 20 and 24 months we recorded the deaths of 4 and respectively 3 patients with NYHA IV congestive heart failure, aged between 68–77 years, with coronary heart disease (3 of 4 and respectively 2 of 3), with myocardial infarction sequelae (2 of 3), with EF 30–50%, with a history of stroke (3 patients) and chronic atrial fibrillation (3 patients).
- at 38 months there were two female fatalities, 67 and 82 years old,  with NYHA class III and IV heart failure at admission, no history of myocardial infarction, both smokers, with ischemic heart disease.
- the last peak of mortality recorded could not be explained by a particular subgroup of patients but by the normal appearance of this curve, which registers an increase among the human population, due to the approach end of the lifetime ‘design’.

Studying this important concept, the hazard function, for the whole group or for each variable taken into account is extremely useful because survival models commonly use hazard function or hazard function logarithm; a parametric model of the hazard function logarithm, based on a multiple linear distribution can be written as h_i_(t)=exp(alpha+a_i_x_i1_+a_2_x_i2_+...+a_n_x_in_)=e^alpha+a_i_x_i1_+a_2_x_i2_+...+a_n_x_in_^

**Figure 8:**
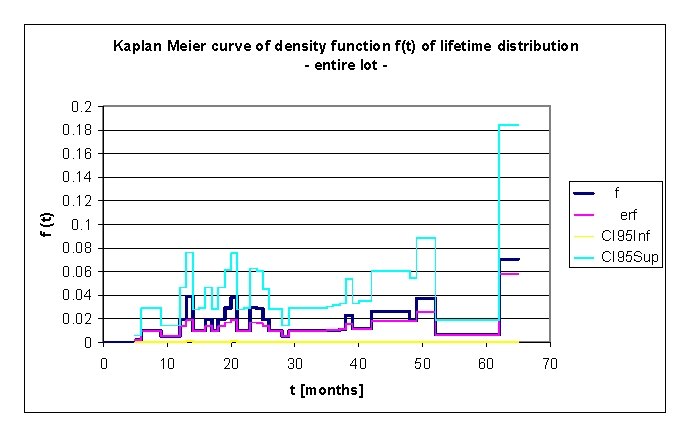
Kaplan Meier curve of density function f(t) of lifetime distribution, for the entire lot

The density function f(t) of lifetime distribution is defined as a continuous random variable f(t)=dF(t)/dt for T.

If f(t) is known, we can calculate the probability that the time of death T will occur before or at the latest at a specified time t, by the relationship F(t)=P(T≤t)=∫ from 0 to t f(u)du. f(t) is sometimes called the event density; it is the rate of death or failure events per unit time.

The explanation of the peaks of mortality recorded and observed in this graph, as in the previous one, is that there are particular subgroups of patients. The explanation in detail is available above.

Then, all variables mentioned above (40 variables integer, real or boolean) were analyzed based on the average, median and specially chosen values. The following elements were studied for each variable: Kaplan Meier curves of survival curve,  for H (t) – cumulative hazard and h (t), F (t) – lifetime distribution function and density function f(t) of lifetime distribution function, MRLT – Mean Residual Life Time, the plot of Ln S (t) vs. t and plot of Ln(H)  vs. Ln (t).

**Figure 9:**
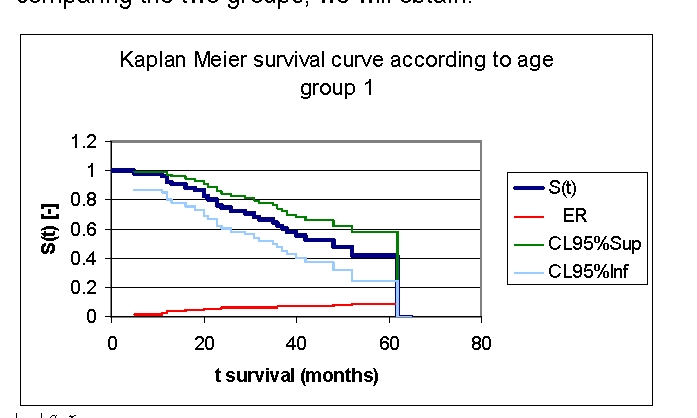
Kaplan Meier survival curve according to age, group 1

**Figure 10:**
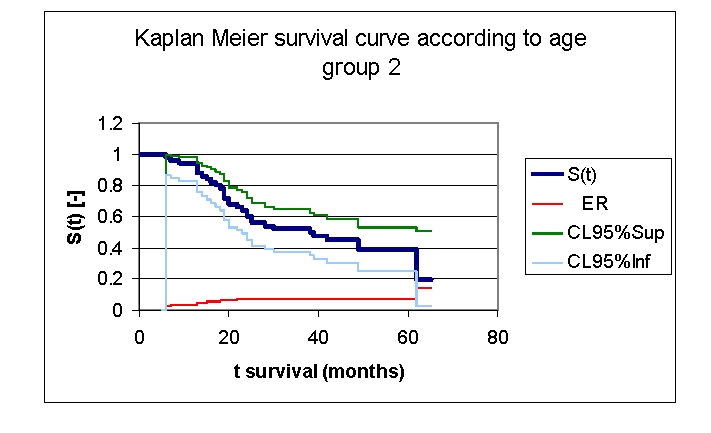
Kaplan Meier survival curve according to age, group 2

**Figure 11:**
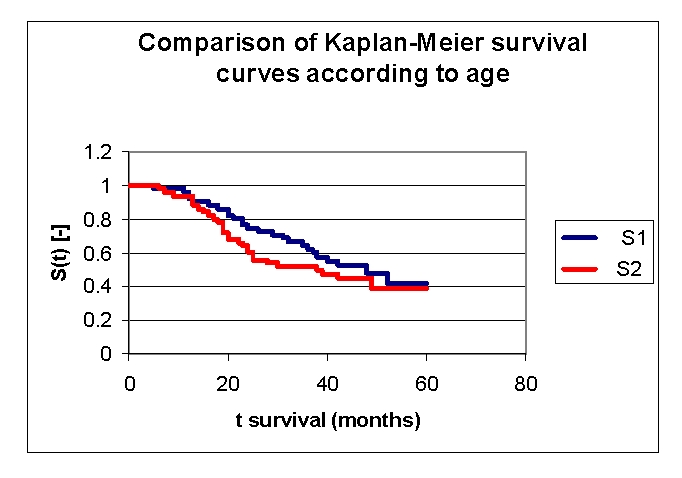
Comparison of Kaplan–Meier survival curves in relation to age

The first group of patients, 40.0 – 71.2 years old, included 33 men, out of whom, 17 have survived, and 18 women, out of whom, 9 have survived. The second group of patients, of 71.2 – 91.0 years old, includes 18 men, out of whom, 6 have survived, and 32 women, out of whom, 14 have survived.

From the graphics above, we remark that the survival curves start separated, after 7 months of follow–up, with net separation after 12 months. What needs to be noted is the proximity of the two curves (without duplication) after 53 months. It is noted, that for a random individual with heart failure, the probability of survival greater than t = 5 years is of 41,76% for the first group of patients and 38,98% for the second group of patients. 

Comparing the two groups we obtained: 92,16% of the patients from the first group of patients survive for more than one year, and, respectively 94% of the patients from the second group of patients survive for more than one year. 76,47% and respectively 62% of the patients survive for more than two years, 64,525% and respectively 52% of the patients survive for more than three years, 47,73% and respectively 44,98% survive for more than four years.

It was noted that 49,74% of the patients from the second group of patients survive for more than 38 months and respectively 50% of the patients from the first group of patients survive for more than 48 months.

**Figure 12:**
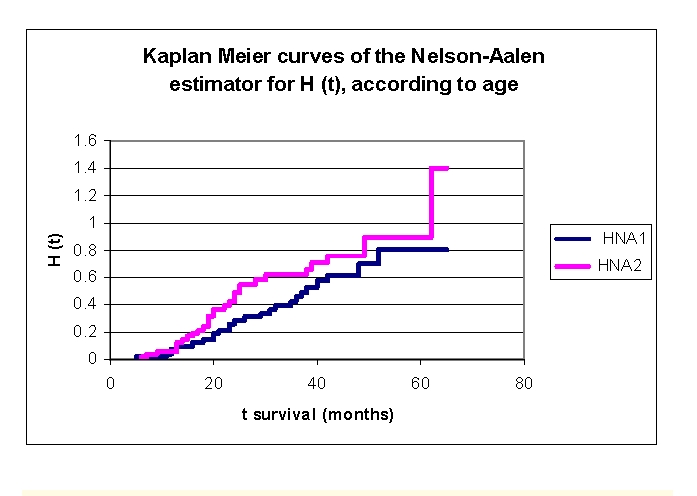
The comparison of the Kaplan–Meier curves, of the Nelson–Aalen estimator for H (t) – cumulative hazard, according to age

We can see from the data obtained that H (t), cumulative hazard, increases from 0.0396 for the patients from first group and respectively 0.0604 for the patients from the second group (at 1 year), 0.2609 and respectively 0.4248 (2 years), 0.4254 and respectively 0.6207 (3 years), 0.6162 and respectively 0.7612 (4 years) to 0.8048 and respectively 0.8945 (5 years). 

**Figure 13:**
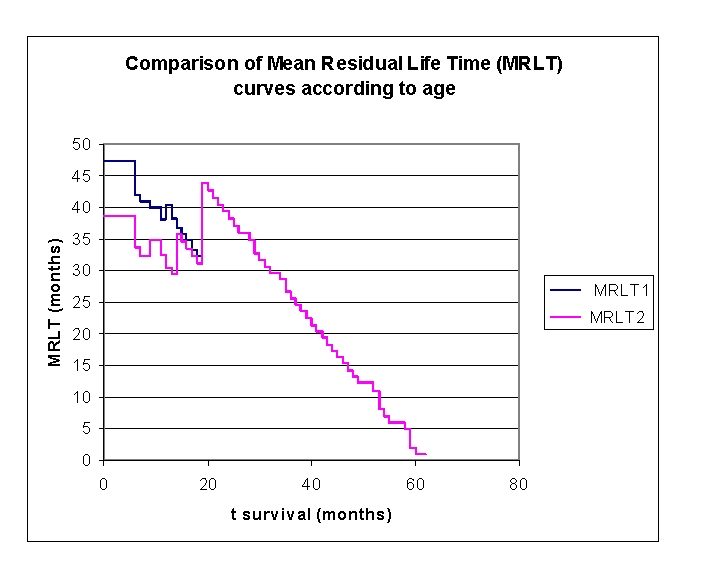
Comparison of MRLT– Mean Residual Life Time curves according to age

The mean residual lifetime (average remaining survival time) in our lot, is of 47.2297 months for the patients from the first group and respectively 38.7001 months for the patients from the second group (at 6 months follow–up), 38.0095 and respectively 32.4573 months (at 12 months follow–up), 33.2169 and respectively 32.2262 months (at 18 months follow–up). 19 months of follow–up date for the first group and are not available/censored. The patients from the second group have 39.3577 months (at 2 years), 26.6651 (at 3 years) and 14.3077 (at 4 years).

**Figure 14:**
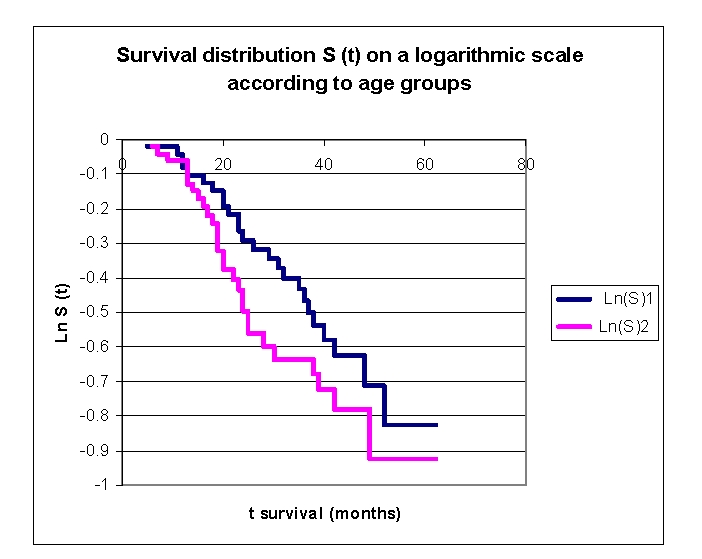
The survival distribution S (t) on a logarithmic scale according to age groups

The survival distribution S (t) on a logarithmic scale in our lot is a straight line for both age groups, these indicating that the exponential distribution may be a reasonable choice for the data.

There were 50 (49,5%) women and 51 men included in our lot. We can divide our lot into two groups: group 1 –women and group 2 – men, subsequently being analyzed separately and in comparison. 

**Figure 15:**
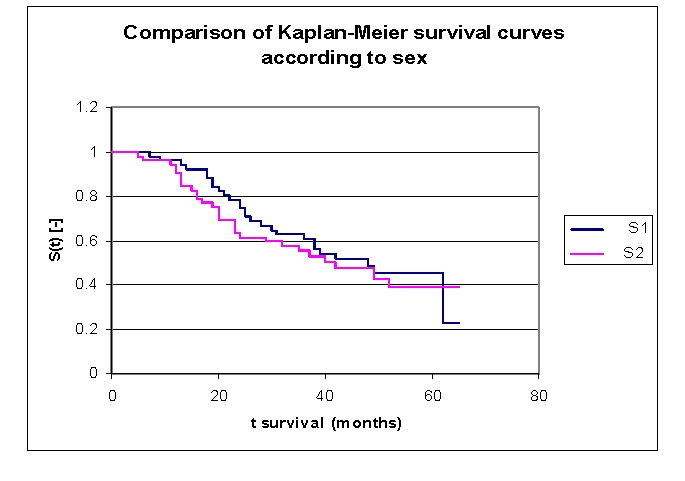
Comparison of Kaplan–Meier survival curves according to sex, group 1 – women and group 2 – men

From the graphics above, we can remark that the survival curves start separating after 5 months of follow–up, with a net separation after 12. The proximity of the two curves can be denoted between 9–11 months. 

Comparing the two groups we obtain the following: 96,04% of the patients from the first group, survive for more than one year, and respectively 94,16% of the patients from the second group, survive for more than one year; 78,41% and respectively 63,3% of the patients, survive for more than two years, 62,72% and respectively 55,36% of the patients survive for more than three years, 51,93% and respectively 47,36% survive for more than four years. It was noted that, for a random individual with heart failure, the probability of survival greater than t = 5 years is of 45,23 for the first group of patients and 38,95 for the second group of patients. 

We observed that 50,32% of the patients from the second group survive for more than 42 months, and respectively 50% of the patients from the first group of patients, survive for more than 48 months. 

**Figure 16:**
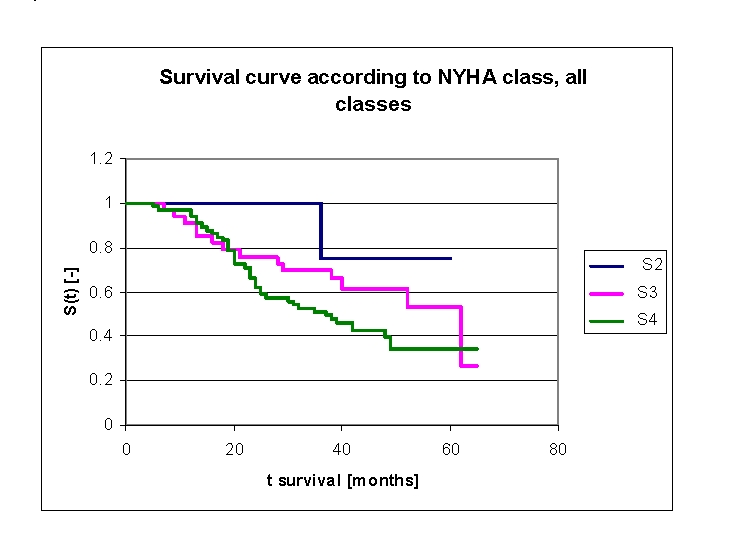
Comparison of Kaplan–Meier survival curves according to NYHA class, group 2 – patients with NYHA class II , group 3 – patients with NYHA class III and respectively group 4 – patients with NYHA class IV at the time of admission to hospital

We included 64 patients (34 men, 30 women) with NYHA class IV in our lot, at the time of admission to hospital, 33 patients (15 men, 18 women) with NYHA class III and respectively 4 patients (2 men, 2 women) with NYHA class II (Table 2).

**Table 2:** Patients' survival according to NYHA class and gender

NYHA class at the time of admission to hospital	Men	Men who died	Men who have survived	Women	Women who died	Women who have survived
NYHA class IV	34	22	12	30	18	12
NYHA class III	15	6	9	18	8	10
NYHA class II	2	0	2	2	1	1

Analyzing the survival curves in the figure above, we remarked that the survival curves start separating after 5 months of follow–up, with a net separation after 20 months (patients with NYHA class IV and III, the 4 patients with NYHA class II have a net positive evolution from the rest of the subjects, but their number is too small for statistical analysis).  The proximity of the two curves (NYHA class IV and III) is denoted between 5–20 months. 

Comparing the groups of patients with NYHA class III and IV we obtained the following: 91,09% of the patients from the group of patients with NYHA class III, survived for more than one year, and respectively 96,9% of the patients from the group of patients with NYHA class IV, survived for more than one year; 75,91% and respectively 66,34% of the patients, survived for more than two years, 69,96% and respectively 51,13% of the patients survived for more than three years, 61,56% and respectively 42,34% survived for more than four years. It was noted that, for a random individual with heart failure, the probability of survival greater than t = 5 years is of 53,36 for the group of patients with NYHA class III and 34,22 for the group of patients with NYHA class IV

We observed that 50% of the patients from the second group of patients (NYHA class IV) survive for more than 37 months and respectively 50% of the patients from the first group of patients (NYHA class III) survive for more than 62 months. 

The analysis of Kaplan–Meier survival curves, according to ejection fraction (FE), can be made according to the mean (Figure 22) and median (Figure 23, more sensitive statistically) rates, or according to special clinically important intervals (Figure 24). We only presented the survival of patients on three intervals, considering the small size of the article.

**Table 3:** Patients' survival according to ejection fraction class and gender

Ejection fraction	Men	Men who died	Men who have survived	Women	Women who died	Women who have survived
group 1 FE 15.0 –30.0%	17	14	3	6	5	1
group 2 FE 30.0–50.0%	32	14	18	36	19	17
group 3 FE 50.0–60.0%	2	0	2	8	3	5

**Figure 17:**
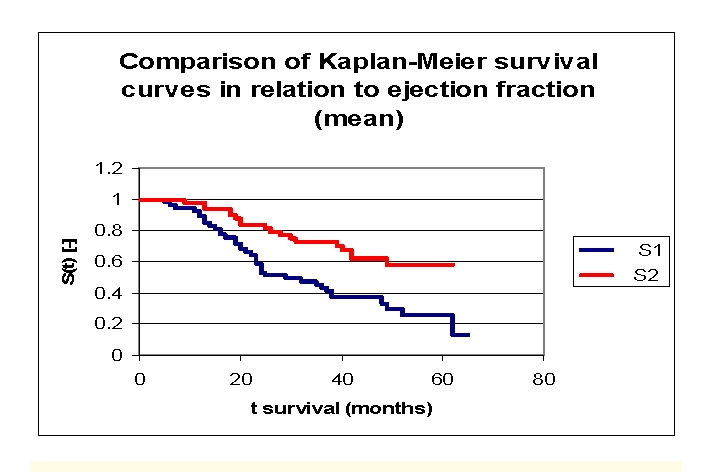
Comparison of Kaplan–Meier survival curves according to the ejection fraction (two groups according to mean, group 1 FE 15.0 – 40.9%, group 2 FE 40.9 – 60.0%)

**Figure 18:**
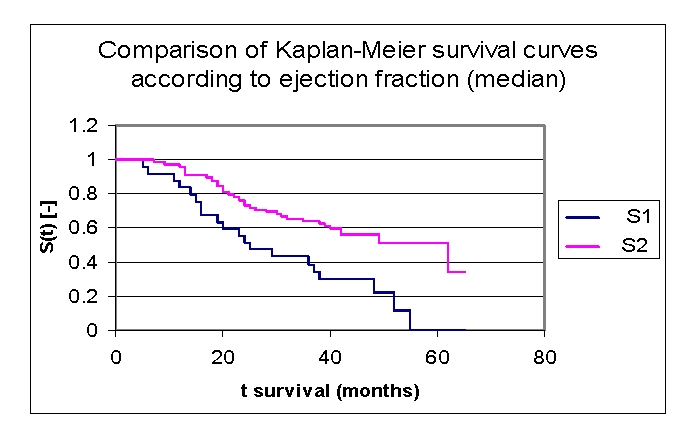
Comparison of Kaplan–Meier survival curves according to the ejection fraction (two groups according to median, group 1 FE 15.0 –33.0%, group 2 FE 33.0– 60.0%)

**Figure 19:**
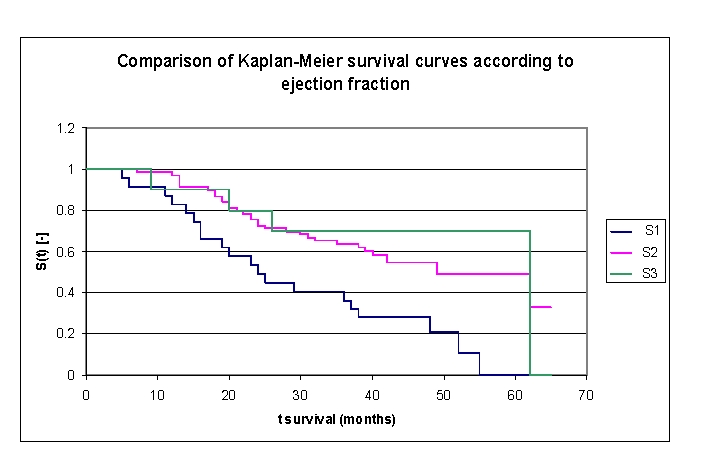
Comparison of Kaplan–Meier survival curves according to the ejection fraction (3 groups, group 1 FE 15.0 – 30.0%, group 2 FE 30.0– 50.0%, group 3 FE 50.0– 60.0%)

While analyzing the survival curves in the figure above, we remarked that the survival curves of patients within FE group 1 and 2, started separating after 5 months of follow–up; the 10 subjects in group 3 had a net positive evolution from the rest of the subjects.

Comparing the three FE groups of patients we obtained the following: 87,14% of the patients from the first group, survived for more than one year, 98,53% of the patients from the second group, survived for more than one year and respectively 90% of the patients from the third group, survived for more than one year; 53,24%, 75,42% and respectively 80% of the patients, survived for more than two years, 40,54% 63,72% and respectively 70% of the patients, survived for more than three years, 28,03%, 54,77% and respectively 70% of the patients,survived for more than four years. It was noted, that for a random individual with heart failure, the probability of survival greater than t = 5 years is of 0 for the group of patients with FE 15.0 – 30.0% and 49,15% for the group of patients with FE 30.0– 50.0%. 

We observed that 53,24% of the patients from the first group, survived for more than 24 months and 54,77% of the patients from the second group, survived for more than 24 months. 

The analysis of Kaplan–Meier survival curves according to left ventricular end diastolic diameter (LVEDD), can be made according to the mean (54.6 mm) and median (65.0 mm, more sensitive statistically) rates, or according to a chosen value (60.0 mm). We will only present the survival of patients according to a chosen value, 60.0 mm of left ventricular end diastolic diameter.

**Figure 20:**
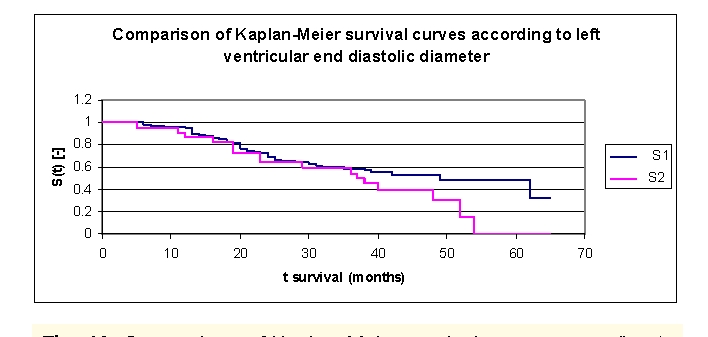
Comparison of Kaplan–Meier survival curves according to left ventricular end diastolic diameter (LVEDD) (2 groups, group 1 LVEDD 37.0  – 60.0 mm, group 2 LVEDD 60.0– 82.0 mm)

While analyzing the survival curves in the figure above, we remarked that the survival curves of the patients within LVEDD group 1 and 2, started separating after 36 months of follow–up.

Comparing the two LVEDD groups of patients we obtained the following: 96,23% of the patients from the first group, survived for more than one year, and respectively 90,91% of the patients from the second group, survived for more than one year; 72,59% and respectively 63,8% of the patients, survived for more than two years, 58,8% and respectively 54,3% of the patients, survived for more than three years, 52,54%, and respectively 39,93% of the patients, survived for more than four years. It was noted, that for a random individual with heart failure, the probability of survival greater than t = 5 years is of 47,97% for the group of patients with LVEDD 37.0 –60.0 mm and 0 for the group of patients with LVEDD 60.0– 82.0 mm. In fact, after 54 months of follow–up no patient in group 2 survived any longer.

We observed that 50% of the patients from the first group, survived for more than 49 months and 50% of the patients from the second group, survived for more than 37 months. 

The analysis of the Kaplan–Meier survival curves, according to the heart rate (HR), can be made according to the mean (100 b/min) or median (64.0 b/min) values. 

**Table 4:** The patients' survival according to heart rate (HR) and gender

Heart rate (HR)	Men	Men who died	Men who have survived	Women	Women who died	Women who have survived
group 1 HR 40.0 –100 b/min	36	19	17	25	13	12
group 2 HR 100–270.0 b/min	15	9	6	25	14	11

**Figure 21:**
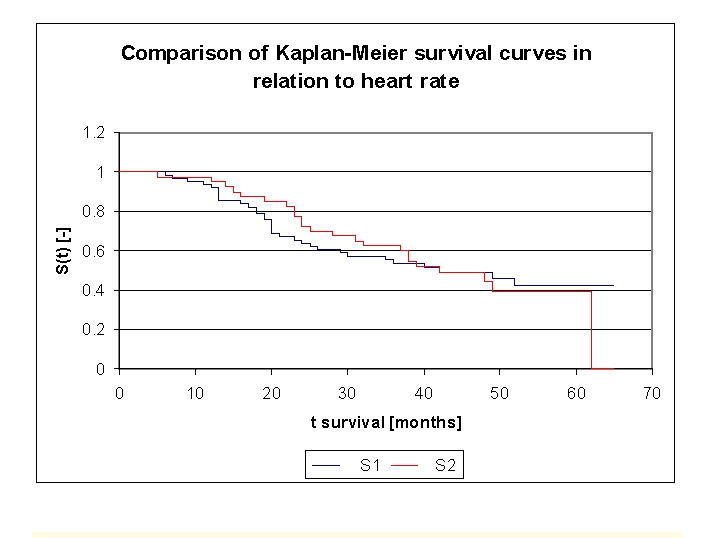
Comparison of Kaplan–Meier survival curves according to heart rate (2 groups, group 1 HR 40.0 –100 b/min, group 2 HR 100 –270.0 b/min).

While analyzing the survival curves in the figure above, we remarked that the two curves start separating after 9 months of follow–up. Comparing the two HR groups of patients we obtain the following: 93,44% of the patients from the first group, survived for more than one year, and respectively 97,5% of the patients from the second group, survived for more than one year; 65,57% and respectively 77,5% of the patients, survived for more than two years, 55,53% and respectively 62,5% of the patients, survived for more than three years. It should be noted that, after 39 months, the two curves showed a similar, close survival of the patients in the two groups. We observed that 50% of the patients from the two groups, survived for more than 42 months.

The analysis of Kaplan–Meier survival curves, according to the systolic blood pressure (TAS), can be made according to the mean (141.5 mmHg) or median (125.0 mmHg) values. We can see the distribution and survival of the patients according to SBP and sex, in Table 5. 

**Table 5:** The patients' survival according to systolic blood pressure and gender

Systolic blood pressure (SBP)	Men	Men who died	Men who have survived	Women	Women who died	Women who have survived
group 1 SBP 70.0–141.5 mmHg	31	24	7	28	18	10
group 2 SBP 141.5–240.0 mmHg	20	4	16	22	9	13

**Figure 22:**
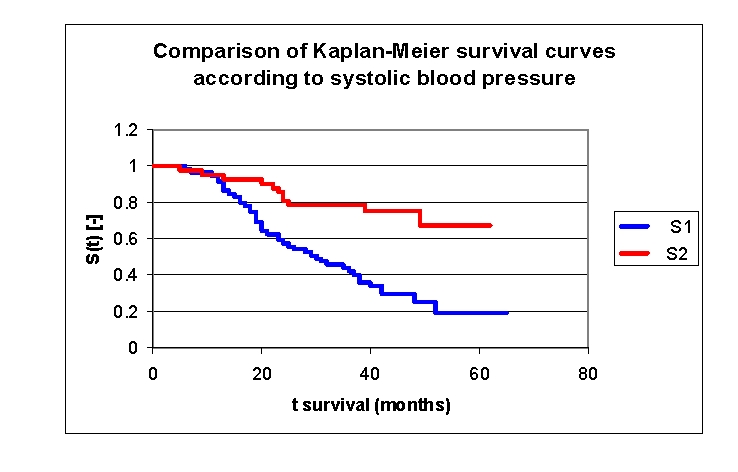
Comparison of Kaplan–Meier survival curves according to the systolic blood pressure (2 groups, group 1 SBP 70.0 – 141.5 mmHg, group 2 SBP 141.5 – 240.0 mmHg).

While analyzing the survival curves in the figure above, we remarked that the two curves start separating after 12 months of follow–up. Comparing the two SBP groups of patients we obtain the following: 94,92% of the patients from the first group, survived for more than one year, and respectively 95,24% of the patients from the second group, survived for more than one year; 59,32%, and respectively 85,71% of the patients, survived for more than two years, 43,86% and respectively 78,57% of the patients, survived for more than three years, 29,78% and respectively 75,77% of the patients, survived for more than four years. It was noted, that for a random individual with heart failure, the probability of survival greater than t = 5 years is of 19,15% for the first group of patients and 67,35% for the second group of patients. 

**Figure 23:**
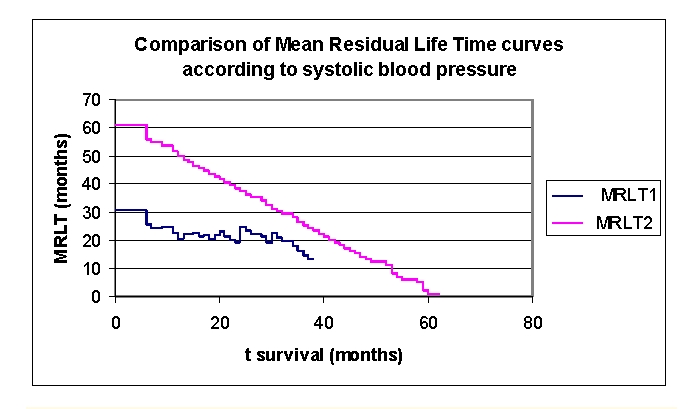
Comparison of mean residual lifetime curves according to the systolic blood pressure (2 groups, group 1 SBP 70.0– 141.5 mmHg, group 2 SBP 141.5 – 240.0 mmHg).

The mean residual lifetime (average remaining survival time) in our lot is of 22.7877 months for the patients from first group and respectively 51.8057 months for the patients from the second group (at 12 months follow–up), 19.1398 and respectively 38.5953 months (at 24 months follow–up), 16.1172 and respectively 26.324 months (at 3 years).

Patients with acute myocardial infarction and patients with a history of myocardial infarction were included in our group. Two boolean variables are considered in the charts below.  

There were 30 patients with a history of myocardial infarction: 19 men and 11 women (fig. 23). Comparing the two groups of patients (group 1 with a history of myocardial infarction, group 2 without a history of myocardial infarction) we obtained the following: 93,44% of the patients from the first group, survived for more than one year, and respectively 97,04% of the patients from the second group, survived for more than one year; 73,52%, and respectively 77,71% of the patients, survived for more than two years, 57,46% and respectively 68,82% of the patients, survived for more than three years, 53,2% and respectively 57,85% of the patients, survived for more than four years. It was noted, that for a random individual with heart failure, the probability of survival greater than t = 5 years is of 45,02% for the first group of patients and 53,52% for the second group of patients. 

**Figure 24:**
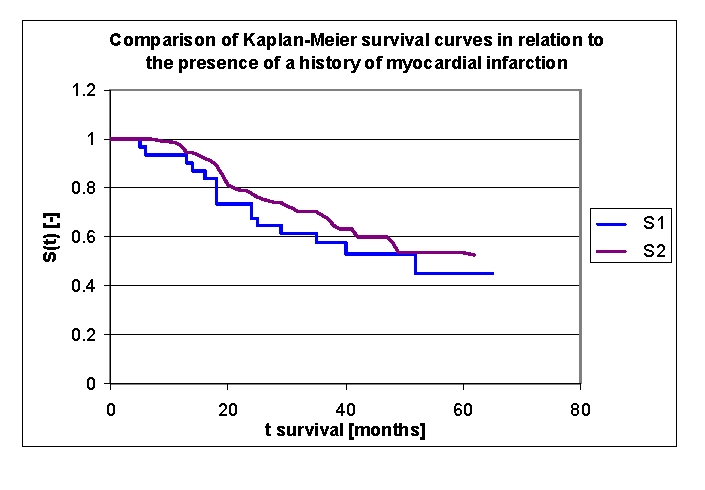
Comparison of Kaplan–Meier survival curves according to a history of myocardial infarction (2 groups, group 1 with a history of myocardial infarction, group 2 without a history of myocardial infarction).

**Figure 25:**
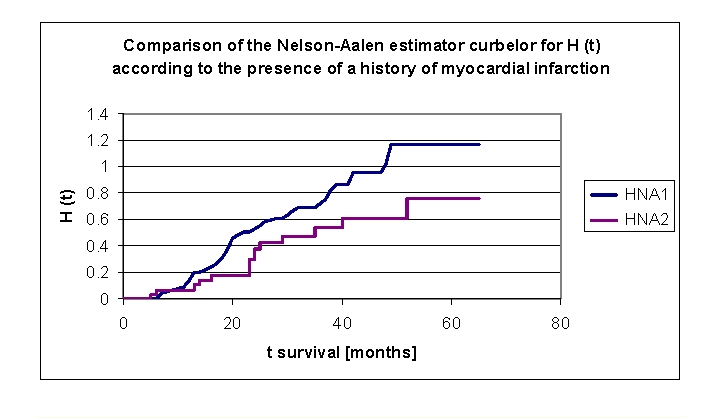
Comparison of Kaplan–Meier curves of the Nelson–Aalen estimator for H (t) –cumulative hazard, according to a history of myocardial infarction (group 1 with a history of myocardial infarction, group 2 without a history of myocardial infarction).

We can see from the data obtained, that the cumulative hazard value is almost double in the first two years for the patients with a history of myocardial infarction, then the difference is significant but slightly smaller: 0.1347 for the patients from first group and respectively 0.0667 for the patients from the second group (at 1 year), 0.5297 and respectively 0.2966 (2 years), 0.7233 and respectively 0.5352 (3 years), 1.0199 and respectively 0.6093 (4 years) to 1.172 and respectively 0.7632 (5 years).

**Figure 26:**
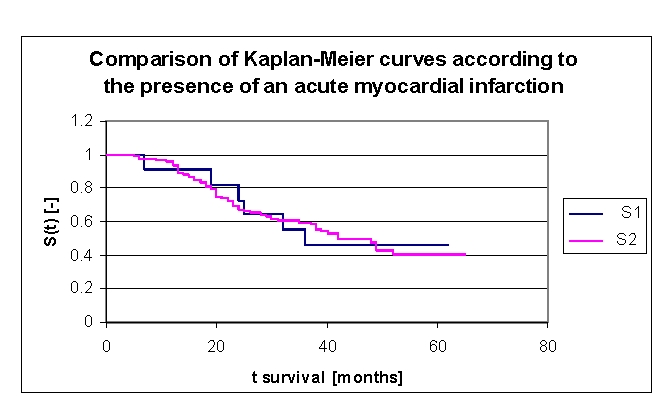
Comparison of Kaplan–Meier survival curves according to the presence of an acute myocardial infarction (group 1 with acute myocardial infarction, group 2 without acute myocardial infarction).

There were 11 patients with an acute myocardial infarction: 2 men and 9 women, an insufficient number for the statistical analysis (Figure 26).

According to the smoking status, the lot was divided into two groups: group 1 – 28 smoking patients (19 men and 9 women) and group 2 –73 non–smoking patients (32 men and 41 women). In up to 38 months of followx2013;up it could be seen that the values of cumulative hazard were similar, subsequently denoting an increase of the cumulative hazard for smoking patients: 0.3259 for first group and respectively 0.3455 for the second group of patients (2 years), 0.6646 and respectively 0.5623 (38 months), 0.9171 and respectively 0.5935 (4 years) and 0.9171 and respectively 0.7906 (5 years).

**Figure 27:**
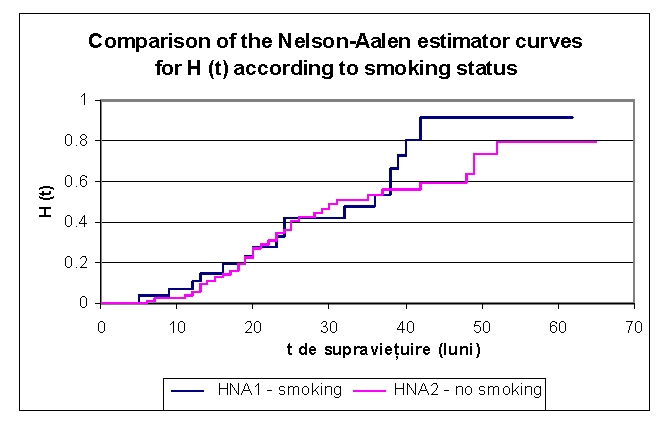
Comparison of Kaplan–Meier curves of the Nelson–Aalen estimator for H (t) – cumulative hazard, according to smoking status

According to the presence of diabetes mellitus, the lot was divided into two groups: group 1 – 36 patients with diabetes mellitus (18 men and 18 women) and group 2 – 65 patients without diabetes mellitus (33 men and 32 women). Comparing the two groups of patients we noticed that: the two survival curves were separating after 9 months; 97,22% of the patients from the first group, survived for more than one year, and respectively 93,92% of the patients from the second group, survived for more than one year; 83,33%, and respectively 63,69% of the patients survived for more than two years, 69,78% and respectively 52,89% of the patients, survived for more than three years, 61,06% and respectively 43,02% of the patients, survived for more than four years. It was noted, that for a random individual with heart failure, the probability of survival greater than t = 5 years was of 55,97% for the first group of patients and 33,36% for the second group of patients. 

**Figure 28:**
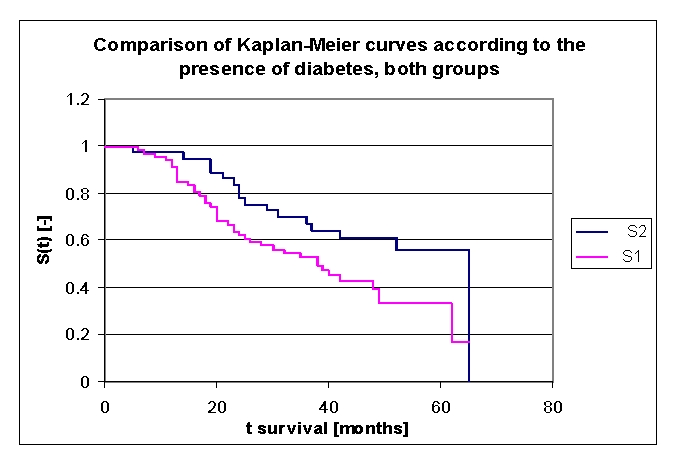
Comparison of Kaplan–Meier survival curves according to the presence of diabetes mellitus (group 1 with diabetes mellitus, group 2 without diabetes mellitus).

**Figure 29:**
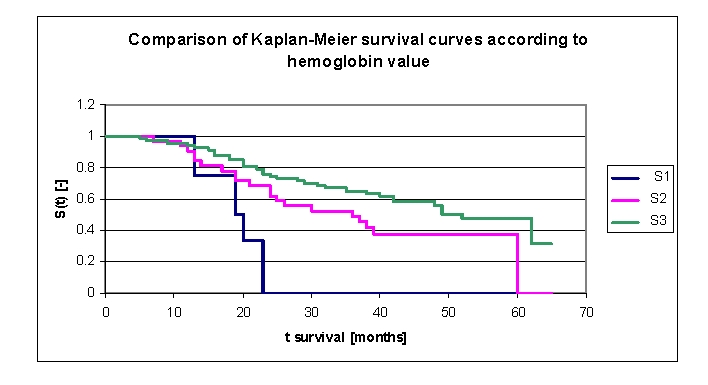
Comparison of Kaplan–Meier survival curves according to hemoglobin value (group 1 Hb 8.6 – 9.5 gr/dl, group 2 Hb 9.5 – 12.5 gr/dl, group 3 Hb 12.5 – 17.3 gr/dl).

Another variable, which was carefully analyzed, was the hemoglobin value at admission; the variable was analyzed based on the average, median and several especially chosen subgroups of values. The lot was divided into three groups: group 1 patients with Hb between 8.6 – 9.5 gr/dl, group 2 patients with Hb between 9.5 – 12.5 gr/dl, group 3 patients with Hb between 12.5 – 17.3 gr/dl (Figure 29). 

The first group consisted of four patients, thus the statistical significance was low. The three survival curves separated after 7 months. Comparing the three groups, we obtained the following: 100% of the patients from the first group, survived for more than one year, and respectively 93,55% of the patients from the second group, survived for more than one year; 95,48 of the patients from the third group, survived for more than one year, 0% , 68,2% , and respectively 75,9% of the patients, survived for more than two years, 52,4% (group 2) and respectively 65,34% (group 3) of the patients, survived for more than three years, 37,75% (group 2)  and respectively 57,99% (group 3), survived for more than four years. It was noted, that for a random individual with heart failure, the probability of survival greater than t = 5 years was of 37,75% for the second group of patients and 47,69% for the third group of patients. 

Other variables carefully analyzed were Na and K values at admission; the variables were analyzed based on the average, median and several especially chosen subgroups of values.

**Figure 30:**
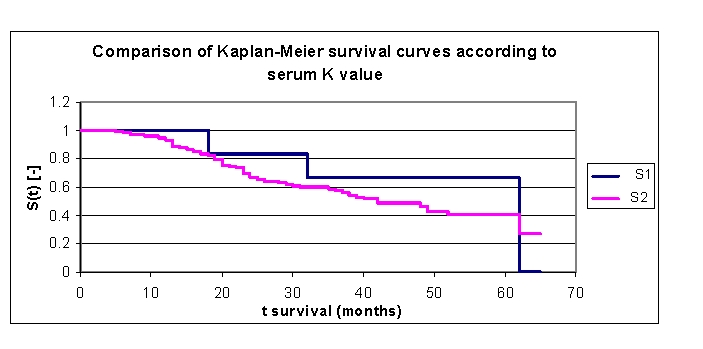
Comparison of Kaplan–Meier survival curves according to serum K value (group 1 K 3.3 – 3.5 mEq/L, group 2 K 3.5 – 5.8 mEq/L).

The lot was divided into two groups according to serum K value: group 1 – patients with K 3.3 –3.5 mEq/L, group 2 – patients with K 3.5 – 5.8 mEq/L. (Figure 30). The first group consisted of six patients, thus the statistical significance was low. The two survival curves separated after 5 months. Comparing the two groups we obtained: 100% of the patients from the first group of patients, survived for more than one year, and respectively 94,78% of the patients from the second group, survived for more than one year; 83,33% , and respectively 70% of the patients, survived for more than two years, 66,67% and respectively 58,61% of the patients, survived for more than three years, 66,67% and respectively 48,8% survived for more than four years. It was noted, that for a random individual with heart failure, the probability of survival greater than t = 5 years was of 66,67% for the first group of patients and 40,62% for the second group of patients

**Figure 31:**
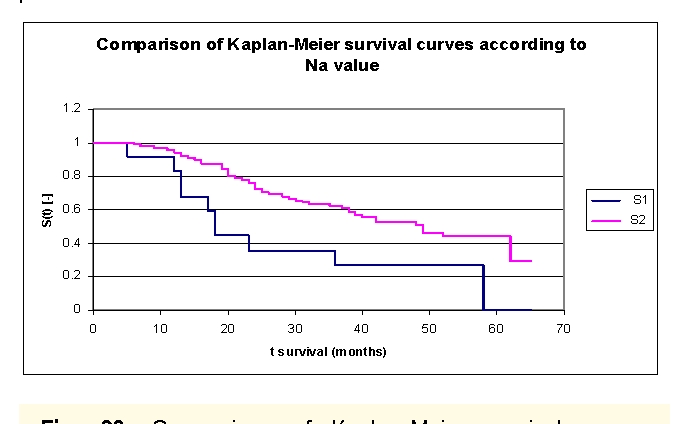
Comparison of Kaplan–Meier survival curves according to serum Na value (group 1 – patients with Na value at admission of 117  – 135 mEq/L, group 2 – patients with Na value at admission of 135 – 148 mEq/L).

The lot was divided into two groups according to serum Na value: group 1 – patients with Na value at admission of 117 – 135 mEq/L, group 2 – patients with Na value at admission of 135 – 148 mEq/L. (Figure 31). The first group consisted of 12 patients and the second had 89 subjects. The two survival curves separated after 5 months. Comparing the two groups we obtained: 91,67% of the patients from the first group of patients, survived for more than one year and respectively 95,54% of the patients from the second group, survived for more than one year; 35,8% and respectively 75,67% of the patients, survived for more than two years, 35,8% and respectively 62,36% of the patients, survived for more than three years, 26,85% and respectively 52,93% of the patients, survived for more than four years. It was noted that for a random individual with heart failure, the probability of survival greater than t = 5 years was of 0% for the first group of patients and 44,02% for the second group of patients. 

Another variable carefully analyzed was the existence of certain classes of drugs in therapy. Due to the limited size of the article, we have only analyzed the patients' evolution according to the use of beta–blockers or angiotensin converting enzyme inhibitors.

The lot was divided into two groups: group 1 – patients with beta–blockers in therapy, group 2 – patients without beta–blockers, see Table 6 – The patients' survival according to the treatment with beta–blockers and gender.

**Table 6:** The patients' survival according to the treatment with beta–blockers and gender

Therapy	Men	Men who died	Men who have survived	Women	Women who died	Women who have survived
group 1 with beta blokers	15	7	8	16	9	7
group 2 without beta blokers	36	21	15	34	18	16

The two survival curves separated after 5 months. Comparing the two groups we obtained the following: 93,55% of the patients from the first group of patients, survived for more than one year, and respectively 95,73% of the patients from the second group, survived for more than one year; 80,87%, and respectively 66,39% of the patients, survived for more than two years, 64,91% and respectively 56,55% of the patients, survived for more than three years, 54,6% and respectively 47,67% of the patients, survived for more than four years. It was noted that for a random individual with heart failure, the probability of survival greater than t = 5 years was of 49,14% for the first group of patients and 47,69% for the second group of patients. 

**Figure 32:**
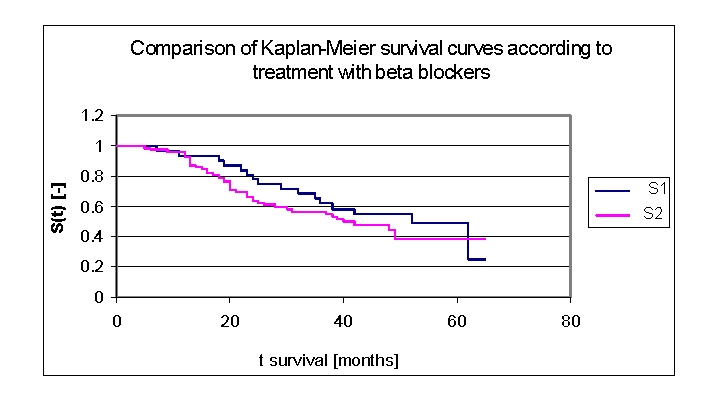
Comparison of Kaplan–Meier survival curves according to treatment with beta–blockers (group 1–patients with beta–blockers in therapy, group 2 – patients without Na beta–blockers in therapy).

We included 68 patients (32 men, 36 women) with angiotensin converting enzyme inhibitors in our lot, and respectively 33 patients (19 men, 14 women) without angiotensin converting enzyme inhibitors in therapy.

The two survival curves started separating after 13 months, with a net difference after 20 months of follow up. Comparing the two groups we obtained the following: 92,73% of the patients from the first group, survived for more than one year, and respectively 93,94% of the patients from the second group, survived for more than one year; 68,21%, and respectively 67,54% of the patients, survived for more than two years, 60,84% and respectively 55,54% of the patients, survived for more than three years, 53,87% and respectively 41,7% of the patients, survived for more than four years. It was noted, that for a random individual with heart failure, the probability of survival greater than t = 5 years was of 42,84% for the first group of patients and 0% for the second group of patients. 

**Figure 33:**
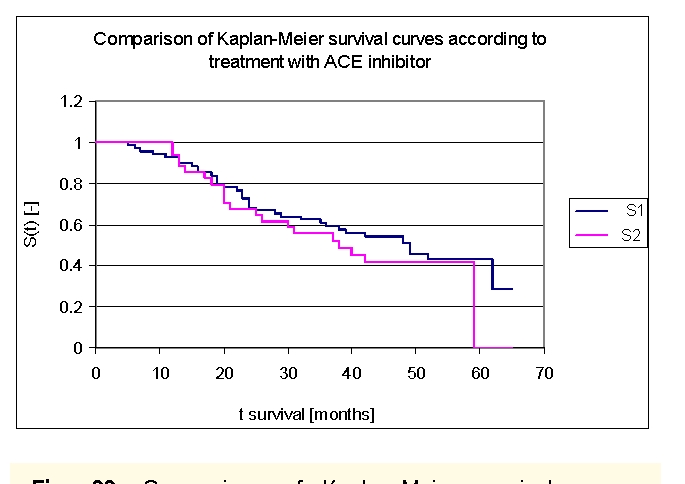
Comparison of Kaplan–Meier survival curves according to treatment with angiotensin converting enzyme (ACE) inhibitors (group 1 – patients with ACE inhibitors in therapy, group 2 – patients without ACE inhibitors in therapy).

In univariate analyses, a series of variables were associated with increased mortality: demographic data (age, sex), etiology (acute or history of myocardial infarction), co morbidity (diabetes, stroke, chronic obstructive pulmonary, cirrhosis, cancer, dementia), smoking status, clinical (systolic blood pressure, heart rate, NYHA class at presentation, systemic or pulmonary congestion,), echocardiographic data (ejection fraction, left ventricular end diastolic diameter), the ECG (QRS> 120ms), laboratory data (hemoglobin, WBC, Na and K value, creatinine, glucose), medication (ACE inhibitors, ß blockers, statins, diuretics dose, antiplatelet, calcium blockers, angiotensin–receptor blocker) and absence of percutaneous transluminal coronary angioplasty. The types of variables are integer, real or boolean. Atrial fibrillation, allopurinol, nitrate, anticoagulant and digitalis were not associated with increased mortality in univariate analysis.

In order to determine the major factors that allow the forecasting survival (or mortality), we used Cox multiple regression model; proportional hazards model [20]. All the data was used in a regression analysis, using Cox proportional–hazards in order to identify the regression coefficients of each variable.

Age and gender were forced into the model as important demographic variables. After many trials the best results obtained were the following: convergence achieved after 17 iterations, for criteria eps2LL = 0.000010; coefficients for each explanatory variable were: 0.0369 (age), –0.0219 (SBP), 0.0570 (Potassium), –0.3124 (sex) and 0.2662 (acute myocardial infarction). Knowing the values of regression coefficients, we had all the elements of the survival function equation. A positive regression coefficient for an explanatory variable meant that the hazard was higher and thus the prognosis worse. Conversely, a negative regression coefficient implies a better prognosis for patients with higher values of that variable. Interpreting the Cox model involves examining the coefficients for each explanatory variable. The impact of adding boolean variables was negative at repeated testing. Accuracy of the model across data sets can be improved by optimizing regression coefficients by repeated testing. 

## Discussion

Our model accurately predicts survival of heart failure patients with the use of commonly obtained clinical characteristics. The score indicated that age, systolic blood pressure, potassium, sex and acute myocardial infarction had independent predictive power. The impact of adding boolean variables was negative at repeated testing. The accuracy of the model can be improved by repeated testing and adding other variables and further investigation is warranted. There were no patients with implantable devices in our study group, so the model did not allow the estimation of the benefit of adding devices to an individual patient's therapeutic regime.

The model easily incorporates obtainable variables that may be available in any hospital, in order to risk stratify patients in a few hours after presentation. Previous heart failure risk models required invasive hemodynamic measurements [21]. 

Our model is derived from a sample of patients hospitalized in an emergency department of cardiology and has not been previously validated among non–hospitalized patients. Another model was developed and validated among outpatients participating in clinical trials, clinical registries or observational studies; therefore, the model may not be generalized to hospitalized patients or those with major life–altering co morbidities. The benefit of knowing the prognosis of these patients with high risk is enormous. The applicability of the model in the general population of patients with heart failure has not been tested. 

## Conclusion

The model developed provides an estimate of survival in patients with acute heart failure using a few variables, easily obtainable in any hospital. There are many applications of such a model, which may estimate the survival of a hospitalized patient with heart failure. The use of this model may facilitate the estimation of vital prognosis, improve compliance, and increase the use of life–saving medical or surgical therapy (pacemakers, implantable defibrillators or transplantation).
